# Hip Fractures in Elderly Individuals Did Not Decrease during the Coronavirus Disease 2019 Pandemic: Insights from the 2015 and 2020 Niigata Prefecture Fragility Hip Fracture Surveys

**DOI:** 10.3390/medicina60040573

**Published:** 2024-03-30

**Authors:** Asami Nozaki, Norio Imai, Yugo Shobugawa, Yoji Horigome, Hayato Suzuki, Hiroyuki Kawashima

**Affiliations:** 1 Division of Orthopedic Surgery, Department of Regenerative and Transplant Medicine, Niigata University Graduate School of Medical and Dental Sciences, 1-754, Asahimachi-Dori, Chuo-Ku, Niigata City 951-8510, Niigata Prefecture, Japan; memi0917memi@yahoo.co.jp (A.N.); hayato-s@yahoo.co.jp (H.S.);; 2 Department of Orthopedic Surgery, Niigata Tsubame Rosai Hospital, Sado 636, Tsubame City 959-1228, Niigata Prefecture, Japan; 3 Division of Comprehensive Musculoskeletal Medicine, Niigata University Graduate School of Medical and Dental Sciences, Niigata City 951-8510, Niigata Prefecture, Japan; 4 Division of International Health, Niigata University Graduate School of Medical and Dental Sciences, Niigata City 951-8510, Niigata Prefecture, Japan

**Keywords:** hip fracture, COVID-19, older adults, Niigata Prefecture

## Abstract

*Background and Objectives*: The incidence of osteoporotic hip fractures in Niigata Prefecture, Japan, has been studied approximately every 5 years since 1985. In 2020, as in previous surveys, a prefecture-wide survey was initiated as planned; however, the global outbreak of the coronavirus disease 2019 (COVID-19) began simultaneously. This study aimed to compare the results of the 2015 and 2020 Niigata Prefecture Fragility Hip Fracture Surveys to determine whether the COVID-19 pandemic affected the occurrence and treatment of proximal femoral fractures throughout Niigata Prefecture. *Materials and Methods*: In this study, data from the 2015 and 2020 Niigata Prefecture Fragility Hip Fracture Surveys were used. Data were obtained from registration forms returned by hospitals and clinics in Niigata Prefecture for patients living therein who were diagnosed with osteoporotic hip fractures over a 1-year period in 2015 and 2020. *Results*: In Niigata Prefecture, the total annual number of fractures increased from 3181 in 2015 to 3369 in 2020, whereas the age-adjusted fracture rate decreased. Regarding the location of the fractures, the proportion of outdoor fractures was lower than that of indoor fractures. The proportion of outdoor fractures decreased over the year as a whole, but in particular, the proportion of outdoor fractures decreased significantly under the issued emergency declarations. The most common reasons for delayed surgery related to COVID-19 were “waiting for PCR results” and “quarantine for fever,” accounting for approximately 1.9% of all causes. *Conclusions*: In Niigata Prefecture, Japan, the effect of the COVID-19 pandemic on the number and rate of fractures was minuscule. The proportion of indoor fractures to outdoor fractures increased during the emergency declaration period. Considering that the number of fragility fractures remains the same during an infectious disease pandemic such as COVID-19, it is necessary to ensure that healthcare resources are available to deal with them.

## 1. Introduction

As life expectancy increases worldwide, fragility fractures due to osteoporosis are becoming more common, causing serious morbidity, reduced quality of life [1], and increased mortality [2]. In Japan, the elderly population increases annually, with the estimated incidence of fragility hip fractures reported to have increased annually from 1986 to 2017 [3,4,5,6], with approximately 200,000 cases occurring annually which is likely to increase further in the future.

Herein, we investigated the incidence of osteoporotic hip fractures in Niigata Prefecture, Japan, at a frequency of approximately once every 5 years since 1985 [7,8,9,10,11,12,13], and the trends in the incidence of osteoporotic hip fractures in Niigata Prefecture have been clarified and reported. In 2020, as in previous surveys, a prefecture-wide survey was initiated as planned [7]; however, the global outbreak of the coronavirus disease 2019 (COVID-19) began simultaneously. The first global case of COVID-19 was reported in Wuhan, China, on 8 December 2019, and the first case in Japan was reported on 16 January 2020. Finally, on 30 January, the World Health Organization issued a worldwide declaration of a public health emergency. There was a shortage of medical services in response to the COVID-19 pandemic worldwide, and the potential impact on the provision of medical care for other diseases increased. The pandemic spread throughout Japan and Niigata Prefecture, and there were concerns about the impact of the shortage of medical care, not only for COVID-19 treatment but also for regular medical care.

Several reports have examined the impact of the COVID-19 pandemic on the occurrence of proximal femoral fractures; however, most studies have involved single hospitals. Amzallag et al. [14] and Malik-Tabassum et al. [15] investigated fragility hip fractures in the elderly during the COVID-19 pandemic and reported that the incidence of fractures and treatment methods were not significantly different compared to the prepandemic period. In this study, it was also estimated that the impact of pandemic activity restrictions on fracture incidence was small, given that fragility fractures in elderly individuals are more likely to occur indoors and with minor external forces. If the incidence of fractures remains unchanged, we consider that the availability of healthcare resources under strain due to the pandemic will be an issue. It is therefore important to investigate the occurrence of fragility fractures during a pandemic in order to prepare for unprecedented situations such as this one. The purpose of this study was to compare the results of the 2015 and 2020 Niigata Prefecture Fragility Hip Fracture Surveys, which examined reports from 73 hospitals and clinics in Niigata Prefecture, to determine whether the COVID-19 pandemic affected the occurrence and treatment of proximal femoral fractures throughout Niigata Prefecture.

## 2. Materials and Methods

This study used data from the 2015 and 2020 Niigata Prefecture Fragility Hip Fracture Surveys. As in all previous surveys, registration form booklets were mailed to all hospitals in Niigata Prefecture, Japan, and all orthopedic clinics in Niigata City, Japan, asking them to complete information on patients seen or admitted for hip fractures. Completed registration forms were returned by post or collected in person [7,8,9,10,11,12,13]. The data collected included all patients who resided in Niigata Prefecture who were diagnosed with osteoporotic hip fractures between 1 January and 31 December 2015, and 1 January and 31 December 2020. Osteoporotic fractures are defined as fractures caused by small external forces, such as standing or falling from a low height. All fractures were classified as either femoral neck or trochanteric. Fractures of the base of the femoral neck and lower part of the femoral condyle were classified as transverse fractures. Twenty-five orthopedic clinics in Niigata City and 48 hospitals in Niigata Prefecture participated in the study. Access to data from these hospitals and clinics was provided for the duration of the study. We excluded patients who did not receive orthopedic treatment; patients living in other prefectures; patients under 60 years of age; those with femoral shaft fractures, pathological fractures, periprosthetic fractures, and fractures due to high-energy trauma; and those who refused to participate in the survey or study. Each hospital completed the registration form and returned it to our hospital [7]. Additionally, ever since the first case of COVID-19 in Niigata Prefecture, COVID-19 testing has been consistently conducted upon hospital admission to prevent the spread of the virus in this region.

The study was designed in accordance with the Declaration of Helsinki and approved by the relevant ethics review board. As this was a retrospective study, the requirement for informed consent was waived.

The study items included the total number of fractures per year and per day, location of the fracture (indoor or outdoor), treatment method (conservative or surgical), preoperative waiting time, and reasons for waiting. The number of fractures per day and the location of the fractures were also compared among three periods. In Niigata Prefecture, we divided the year into three periods: the period during which a state of emergency was declared, defined as the “emergency declaration period” (16 April–21 May 2020; 28 days); the period during which Niigata Prefecture issued its own warnings and advisories in addition to declaring a state of emergency, defined as the “issued period” (16 April–21 May, 31 July–8 September, and 11 November–31 December 2020; 117 days); and the period during which no declarations were announced, defined as the “normal period.” The number of fractures in 2015 was calculated from the number of fractures in the same period in 2020. Statistical comparisons were performed using a *t*-test for the number of fractures per day and a chi-square test for the location of fractures. Statistical significance was set at *p* < 0.05. These statistical analyses were performed using Microsoft Excel 2013 (Microsoft Corp., Redmond, WA, USA).

## 3. Results

In Niigata Prefecture, the total annual number of fractures increased slightly from 3181 in 2015 to 3369 in 2020. The total population, population over 65 years of age, percentage of population > 65 years of age (%), and incidence of fracture for each survey year are shown in Table 1. This study was based on data from the Niigata Prefecture Fragility Fracture Survey, and when age-adjusted to the population over 60 years of age in 1985, when the survey began, the fracture rate was 231.05 fracture/100,000 persons/year in 2015 and 222.79 fracture/100,000 persons/year in 2020. We have previously reported a slight decrease in age-adjusted fracture rates [7]. The number of fractures per day was compared for the three periods, the normal, issued, and emergency declaration periods, as well as for the entire year (Table 2), and there were no significant differences across the periods.

In Niigata Prefecture, we divided the year into three periods: the period during which a state of emergency was declared which we called the “emergency declaration period” (16 April 16–21 May 2020; 28 days); the period during which Niigata Prefecture issued its own warnings and advisories in addition to declaring a state of emergency which we called the “issued period” (16 April–21 May, 31 July–8 September, and 11 November–31 December 2020; 117 days); and the period during which no declarations were announced which we called the “normal period.” The number of fractures in 2015 was calculated from the number of fractures in the same period in 2020.

**Figure 1:**
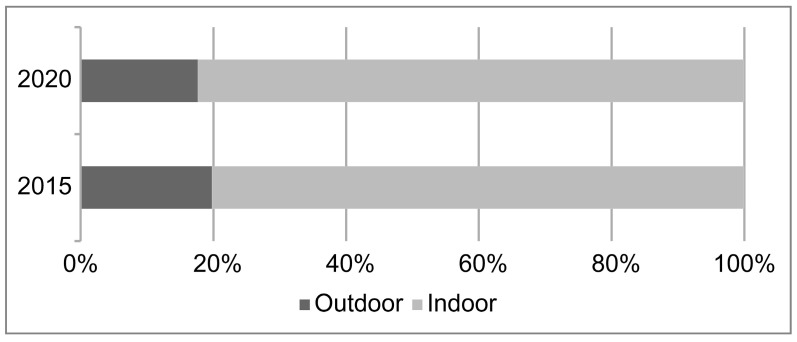
Location of the fracture, indicating that the proportion of outdoor fractures decreased compared to that of indoor fractures (χ^2^[1] = 5.879; *p* < 0.05).

Regarding the location of the fracture, the proportion of fractures which occurred outdoors decreased compared to that of fractures which occurred indoors (χ^2^[1] = 5.879; *p* < 0.05) (Figure 1). The outdoor proportion decreased over the year, but in particular, the outdoor proportion decreased significantly during the declaration (issued and emergency declaration periods) (Table 3).

The proportion of outdoor fractures decreased over the year as a whole, but the proportion of fractures in outdoor spaces decreased significantly under the declaration (issued and emergency declarations).

The ratio of surgical therapy to conservative therapy was 8:1 in 2015 and 12:1 in 2020, demonstrating an increase in the proportion of patients who underwent surgical therapy in 2020. The types of surgery in this study were mostly osteosynthesis and hemiarthroplasty/total hip arthroplasty. The type of surgery was selected by the surgeon according to the respective fracture type. In the 2020 survey, the choice of conservative therapy was based on factors such as poor general health (47%), patient wish against surgery (29%), and fractures not requiring surgery (17%). The influence of COVID-19 on patients’ decision to choose conservative therapy remains unknown. We reported that 49.6% of the surgeries were performed within 48 h. The reasons for waiting for surgery or for surgical delay beyond 48 h were classified as follows: (1) “Holiday,” a holiday occurred between the injury and surgery; (2) “Operating room and anesthesiology,” convenience related to operating room availability and the anesthesiology department; (3) “Orthopedic surgery,” at convenience of the orthopedic department; (4) “Medical comorbidity,” medical complications requiring treatment or examination before surgery; (5) “Delayed consultation,” where there was delay of several days noted at the time of consultation; and (6) “Other,” causes that do not fall under the aforementioned reasons, such as family reasons. These details are described in Table 4 [7]. COVID-19-related causes were categorized as “Other,” including “waiting for PCR results” and “quarantine for fever,” accounting for approximately 1.9% of all cases, including two patients who had COVID-19 and were isolated for 8 days before surgery.

COVID-19-related causes were categorized as “Other,” including “waiting for PCR results” and “quarantine for fever,” accounting for approximately 1.9% of all cases.

## 4. Discussion

The number of fractures was not significantly different when comparing the respective time periods of 2015 and 2020, nor when comparing the normal and emergency declaration periods within one year of 2020, showing little impact of the COVID-19 pandemic.

Among the published reports, those from a single hospital reported that the number of hip fractures was the same, but the total number of fractures including other sites decreased [16], and the number of wrist fractures was the same. However, the number of fractures due to sports and traffic accidents decreased and that of fractures in elderly patients increased [17]. On a larger scale, a report that aggregated hip fractures across Charlotte, NC, United States, found a 53% reduction in the number of fractures (from 16,068 to 7498 fractures) during the lockdown period (1 March to 30 April 2020) [18]. However, a report from six hospitals in England found that the number of hip fractures was comparable (386 and 381 fractures in 2019 and 2020, respectively) during the first lockdown period in 2020 (23 March to 11 May) [19]. As in this study, there are several reports of similar numbers of proximal femur fractures, suggesting that a global pandemic of an infectious disease, such as the COVID-19 pandemic, may not change the number of fragility fractures in the elderly. Even if there is a shortage of medical services, it is also necessary to ensure medical care to deal with fragility fractures in the elderly under all circumstances.

Regarding the proportion of indoor to outdoor locations of fracture occurrence, fragility fractures in older adults were originally fractures that occurred mostly indoors, and the proportion of indoor occurrences increased in the 2020 survey. In addition, the proportion of indoor fractures increased further during the emergency declaration and issuance periods. Poggetti et al. reported that many fractures occurred indoors [17]. This could be due to a decrease in the proportion of outdoor occurrences due to a decrease in the frequency of going out as a result of the declaration, as well as an increase in easy falls and a worsening of osteoporosis due to a decrease in activity. There have been reports of the discontinuation of osteoporosis treatment during the COVID-19 lockdown period [20] and the risk of osteoporosis exacerbation associated with reduced physical activity, daylight hours, and COVID-19 infection [21]. From the above, the importance of fall and osteoporosis prevention has become more apparent, and it is necessary to promote training to improve locomotive syndromes, as recommended by the Japanese Orthopedic Association [22], in addition to improving the home environment through universal design.

Regarding the choice of treatment method, the proportion of surgical treatments increased in 2020 compared to that in 2015, and the COVID-19-related reasons for increased surgical waiting times were low at 1.9%, suggesting that the COVID-19 pandemic had a small impact on the choice of treatment method and treatment timing. However, whether the patients who opted for conservative treatment in this study included COVID-19-positive patients remains unknown, and the impact of post-operative complications and other factors in COVID-19-infected patients remains to be elucidated. In fact, a report from the United States stated that, although there was no significant impact on surgical selection in fracture patients as a whole, the number of days to surgery was increased, conservative treatment was more frequently chosen in COVID-19-positive patients, and mortality rates were very high [18]. This study used data from the 2020 survey of proximal femur fractures in the elderly, which was conducted without anticipating the global pandemic of COVID-19, and the extraction of information about whether a patient had COVID-19 or not was based on entries in the complications section and interviews at each hospital. A limitation of this study was that cases of COVID-19 complications might have been missed. As the data were originally used for an epidemiological survey of hip fractures in Niigata Prefecture, it was possible that COVID-19 complications may not have been listed when the survey sheets were submitted by each hospital. As the data in this study were collected anonymously, the number of patients with repeated fractures per year is unknown, which is another limitation of this study. Furthermore, it is unclear from the data used in this study whether there were any cases in which the COVID-19 epidemic forced the discontinuation of osteoporosis medications, and the possibility that the discontinuation of osteoporosis medications affected fractures is also unknown, and we consider this to be a limitation.

## 5. Conclusions

In Niigata Prefecture, Japan, the effect of the COVID-19 pandemic on the number and rate of fractures was small. The proportion of indoor to outdoor fractures increased during the emergency declaration period. Considering that the number of fragility fractures remained the same during an infectious disease pandemic, such as the COVID-19 pandemic, it is necessary to ensure that healthcare resources are available to deal with them.

## Figures and Tables

**Table 1:** Total population, population over 65 years of age in Niigata Prefecture, and the standardized incidence rates were adjusted to the proportion of the Japanese population aged ≥60 years in 1985.

	2015	2020
Total population in Niigata prefecture	2,303,052	2,198,210
Population over 65 years of age in Niigata prefecture (male/female)	684,758 (291,214/393,544)	721,618 (312,009/409,609)
Percentage of population > 65 years of age in Niigata prefecture (%)	30.0	32.8
Standardized Incidence (fracture/100,000 persons/year)	367.1	388.3

**Table 2:** The number of fragility hip fractures in each of the three periods in Niigata Prefecture.

	2015	2020	*p* Value
Number of hip fractures	3214	3369	
Number of hip fractures per day			
Whole	8.8	9.2	0.053
Normal period	8.9	9.2	0.34
Issued period	8.6	9.2	0.18
Emergency declaration period	9.4	9.6	0.88

**Table 3:** The indoor–outdoor incidence of fracture occurrence in the three periods.

	2015	2020	*p* Value
Whole	4.1:1	4.8:1	0.01
Normal period	4.4:1	4.7:1	0.49
Issued period	3.2:1	5.1:1	<0.01
Emergency declaration period	3.2:1	6.4:1	<0.01

**Table 4:** The timing of surgery and reasons for delays in surgery beyond 48 h for hip fractures among residents of Niigata Prefecture in 2020.

	Number	Percentage
Timing of surgery		
≤48 h	1481	49.6
>48 h	1504	50.4
Reason for delay in surgery		
Holiday	608	40.4
Operating room and anesthesiology	433	23.0
Orthopedic surgery	143	7.6
Medical comorbidity	241	12.8
Delayed consultation	379	20.1
Other	81	4.3
COVID-19 related	29	1.9

Abbreviations: COVID-19, coronavirus disease 2019.

## Data Availability

The original contributions presented in the study are included in the article material, further inquiries can be directed to the corresponding author.
